# The actin regulator zyxin reinforces airway smooth muscle and accumulates in airways of fatal asthmatics

**DOI:** 10.1371/journal.pone.0171728

**Published:** 2017-03-09

**Authors:** Sonia R. Rosner, Christopher D. Pascoe, Elizabeth Blankman, Christopher C. Jensen, Ramaswamy Krishnan, Alan L. James, John G. Elliot, Francis H. Green, Jeffrey C. Liu, Chun Y. Seow, Jin-Ah Park, Mary C. Beckerle, Peter D. Paré, Jeffrey J. Fredberg, Mark A. Smith

**Affiliations:** 1 Department of Environmental Health, Harvard School of Public Health, Boston, Massachusetts, United States of America; 2 University of British Columbia Center for Heart Lung Innovation, St Paul Hospital, Vancouver, British Columbia, Canada; 3 Huntsman Cancer Institute, University of Utah, Salt Lake City, Utah, United States of America; 4 Center for Vascular Biology Research, Beth Israel Deaconess Medical Center, Boston, Massachusetts, United States of America; 5 Department of Pulmonary Physiology and Sleep Medicine, West Australian Sleep Disorders Research Institute, Sir Charles Gairdner Hospital, Nedlands, West Australia, Australia; 6 School of Medicine and Pharmacology, University of Western Australia, Perth, Western Australia, Australia; 7 Department of Pathology and Laboratory Medicine, University of Calgary, Calgary, Alberta, Canada; 8 Department of Biology, University of Utah, Salt Lake City, Utah, United States of America; Cinvestav-IPN, MEXICO

## Abstract

Bronchospasm induced in non-asthmatic human subjects can be easily reversed by a deep inspiration (DI) whereas bronchospasm that occurs spontaneously in asthmatic subjects cannot. This physiological effect of a DI has been attributed to the manner in which a DI causes airway smooth muscle (ASM) cells to stretch, but underlying molecular mechanisms–and their failure in asthma–remain obscure. Using cells and tissues from wild type and zyxin^-/-^ mice we report responses to a transient stretch of physiologic magnitude and duration. At the level of the cytoskeleton, zyxin facilitated repair at sites of stress fiber fragmentation. At the level of the isolated ASM cell, zyxin facilitated recovery of contractile force. Finally, at the level of the small airway embedded with a precision cut lung slice, zyxin slowed airway dilation. Thus, at each level zyxin stabilized ASM structure and contractile properties at current muscle length. Furthermore, when we examined tissue samples from humans who died as the result of an asthma attack, we found increased accumulation of zyxin compared with non-asthmatics and asthmatics who died of other causes. Together, these data suggest a biophysical role for zyxin in fatal asthma.

## Introduction

Of all known bronchodilators, among the most effective are the deep inspirations (DIs) and sighs that occur spontaneously in humans roughly once every 6 minutes [1–8]. In the human, moreover, DIs suppress agonist-induced bronchospasm as well as exercise-induced bronchospasm [2, 9–11]. In the living guinea pig, similarly, imposed DIs suppress hyperpnea-induced bronchospasm [12]. In addition to dilating bronchospastic airways, DIs taken before administration of a constricting agonist attenuate subsequent airway narrowing, a phenomenon termed DI-induced bronchoprotection [13]. Simply put, in the healthy lung breathing facilitates breathing [14]. During a spontaneous asthmatic attack, however, this favorable dynamic becomes substantially attenuated or even reversed [1–3, 14, 15].

To explain the salutary effects of a DI a variety of reductionist approaches have been undertaken using both theory and *in vitro* experimentation. To simulate a DI, investigators typically impose a tissue stretch that is comparable in magnitude and in timing to that expected physiologically *in vivo*. These approaches have spanned the levels of the molecular acto-myosin interaction [16–20], the isolated airway smooth muscle (ASM) cell [21, 22], the isolated ASM strip [6, 17, 23, 24], and the small airway integrated within the precision cut lung slice[15, 25–30]. These approaches have yielded results that are largely consistent with one another and also compatible with observations made in intact animals and humans *in vivo*. By contrast, others have used the experimental preparation of the isolated segment of central airway to show little or no bronchodilatory effect in response to imposed ASM stretch [31–33]; the reasons remain unclear for this striking discrepancy between results obtained using this particular preparation versus the body of literature described above. Nevertheless, the overwhelming weight of evidence *in vivo*, *in vitro*, and *in silico* supports the proposition that during induced bronchospasm a DI produces marked bronchodilation that increases with increasing magnitude of the applied stretch, and that this bronchodilation is attributable to stretch-induced reduction in contractile forces generated by airway smooth muscle.

During the spontaneous bronchospasm that is a cardinal feature of asthma, a DI typically fails to produce bronchodilation, however [1–3]. The mechanisms accounting for this failure remain unclear. This failure has been attributed in varying degrees to a wide variety of sources including: decreased tethering of the airway wall to the surrounding lung parenchyma [23, 34, 35], thickened connective tissue layers [34] [35] and increased ASM tissue mass [23] [14], all of which are thought to protect the ASM from stretch during a DI and thus permit ASM to remain in a shortened and stiffened contractile state. Additional hypotheses to explain the observations include ASM adaptation [36, 37]; increased ASM shortening velocity or passive stiffness [38] [39]; and a defective response to stretch at the level of asthmatic airway smooth muscle itself [40].

Here we suggest a specific molecular mechanism that extends these ideas and helps to unify and explain them. A candidate regulator of ASM contraction is the LIM-domain protein zyxin. Zyxin has never before been implicated in airway dysfunction, but is known to be a highly dynamic and mechano-responsive regulator of the actin cytoskeleton that localizes to focal adhesions and accumulates rapidly at sites of spontaneous acute stress fiber strain [41–43]. Zyxin-mediated stress fiber repair occurs on a timescale comparable to that of re-contraction of ASM after stretch [21, 43] suggesting that zyxin might play a role in regulating key mechanical properties of ASM and its response to a DI. To address this issue, we studied the physiology of cells and tissues from wild type and zyxin^-/-^ mice. We found that in response to a transient stretch of physiologic magnitude and duration, zyxin repairs cyto-architecture at localized regions of stress fiber fragmentation, facilitates recovery of contractile force generated by the isolated mouse ASM (MASM) cell, and slows dilation of the small airway embedded within a precision cut lung slice (PCLS). At the levels of cytoskeleton, isolated cell, and integrated airway tissue, zyxin is thus seen to stabilize ASM structure and contractile properties at current muscle length. To assess the relevance of this finding to humans, we then compared the expression of zyxin in ASM obtained from bronchi of non-asthmatic subjects to that from fatal and non-fatal asthmatic subjects. Importantly, we found increased abundance of zyxin in the ASM of fatal asthmatics. Assessment of the role of zyxin in the regulation of murine airway dynamics in the PCLS combined with the differential abundance of zyxin protein in fatal asthmatics points to pathological overexpression of zyxin as a potential explanation of the failure of a DI to dilate bronchial airways in fatal asthma.

## Materials and methods

### Animals

All animal procedures were approved by the University of Utah Institutional Animal Care and Use Committee for this specific study. Animals were backcrossed nine generations into the inbred C57BL/6 line (Charles River Laboratories) to ensure genetic homogeneity except at the zyxin locus [44]. Lungs were obtained from four wild type and three zyxin^-/-^ C57BL/6 mice at 16-week of age. Mice were briefly anesthetized with Isoflurane and sacrificed via cervical dislocation. [44].

### Cell lines and cell culture

Derivation and immortalization of embryonic fibroblasts from zyxin^-/-^ mice was described previously[45]. Briefly, these zyxin^-/-^ fibroblasts were stably rescued with N-terminally tagged zyxin by viral infection followed by FACS sorting to select cells expressing GFP-tagged zyxin[46]. To image actin stress fibers, these MEFs were subsequently transfected with Lifeact-mApple. As described previously[47], primary airway smooth muscle cells were isolated from the tracheas of wild type and zyxin^-/-^ mice and cultured on soft polyacrylamide gels without further passaging. Human ASM cells were obtained from R. Panettieri (University of Pennsylvania) and cultured in Ham’s Nutrient Mixture F-12 medium supplemented with 10% fetal bovine serum (FBS), 100 U ml^-1^ penicillin, 100 μg ml^-1^ streptomycin, 200μg ml^-1^ amphotericin B, 12 mM NaOH, 1.6 mM CaCl_2_, 2 mM l-glutamine and 25 mM HEPES.

### Live-cell imaging for protein dynamics studies and analysis

Cells for live imaging were plated in DMEM/F12 media lacking phenol red. Imaging was performed on an inverted Nikon T*i*-E (Nikon Instruments) equipped with either a Nikon 20X Plan Apo NA .75 dry, or a Nikon 40X Apo LWD NA 1.15 water immersion lens. This system also utilized an Andor spinning disk confocal (Andor Technology) and an Andor Ixon 885 EMCCD camera. Automation and image capture utilized Andor IQ software running on a Hewlett Packard workstation.

### Spontaneous bead movement tracking and analysis

As described previously [48], spontaneous bead motions are recorded to measure cytoskeletal remodeling. The overall rate of remodeling was quantified by the mean square displacement (MSD) over changes in time.

### RNA interference

HASM cells were plated overnight and then transfected with either a pool of 4 non-coding siRNAs (ON-TARGETplus Non-targeting Pool Cat #D-001810-10-05, Dharmacon, PA, USA) or 4 zyxin-targeted siRNAs (ON-TARGETplus SMARTpool—Human ZYX Cat #L-016734-00-0005, Dharmacon) at a concentration of 25 nM using a transfection reagent at a concentration of 0.2% by volume (DhamaFECT 1 siRNA Transfection Reagent Cat #T-2001-02, Dharmacon). To test the efficiency of zyxin knock down, zyxin mRNA expression was determined by qPCR at 48 hours and zyxin protein was confirmed by western blot at 72 hours. All experiments were performed at 72 hours to ensure adequate protein knockdown.

### Traction force microscopy and stretch

Imaging of MEFs was performed on PDMS gels coupled to 200 nm fluorescent beads and fibronectin. Stretch was applied using an annular condenser mounted indenter, as described in Krishnan et al.[22]. Following each experiment, an additional stretch was applied to verify the magnitude of stretch.

Primary ASM cells were plated on 4 kPa polyacrylamide gels embedded with 200 nm fluorescent beads and allowed to adhere and stabilize over 96 hours (48 hours for MEFs). As described previously in Butler et al.[49], the traction field was calculated from a displacement vector map of changes in bead positions using Fourier transform traction cytometry. This field was used to calculate the net contractile moment of the cell[49]. Stretch experiments were conducted as follows: baseline images of the cell and beads were recorded and then homogeneous isotropic stretch of between 5% and 10% strain was applied. Bead images were collected every 10 seconds for 10 minutes following stretch (or for 15 minutes in the case of the multiple stretch experiment), followed by a final reference image of the gel after the cell’s removal by trypsin. Again, stretch was applied using an annular condenser mounted indenter, as described in Krishnan et al.[22].

### Precision cut lung slice preparation and culture

PCLS were prepared as described previously[50, 51]. Following tracheotomy, the mouse lungs were insufflated with 1% low-melting point agarose in HBSS and placed in cold HBSS (Corning Life Sciences, MA, USA). After the agarose had formed a gel, the two largest lobes were separated and then sectioned into 250 μm thick slices using a Precisionary Instruments VF-300 tissue slicer. Lung slices were cultured in 1:1 DMEM/F-12 supplemented with penicillin, streptomycin, kanamycin, and amphotericin B (Invitrogen, MA, USA). Media was changed once an hour for the first 4 hours. As described in Rosner et al.[52], the PCLS were then slowly frozen at a rate of 1°C/minute in culture media supplemented with 10% DMSO. For the dose-response and stretch experiments, the PCLS were rapidly thawed in a 37°C water bath and cultured in fresh media for at least 2 hours before experiments were performed.

### Methacholine dose-response and stretch of PCLS

Using a ring of nylon mesh embedded in a PDMS ring, each PCLS was secured in place on a 4 kPa polyacrylamide gel in a standard 6 well culture plate. The multi-well plate was mounted on a computer controlled translation stage and imaged using an inverted microscope (Leica Microsystems, DMI6000B, with adaptive focus, motorized stage, and stage incubator, IL, USA). Intact airways were treated with 10^−7^ M, 10^−6^ M, 10^−5^ M, and 10^−4^ M methacholine (MCh, Sigma Aldrich, St. Louis, MO). Subsets of these airways were stretched for 5 minutes with an annular indenter as described in Lavoie et al. [15]. Briefly, an annular indenter was lowered until it compressed the gel on which the PCLS lies, causing the confined gel to bulge and apply a radial stretch to the lung tissue within the indenter (Fig B in S1 File). Each airway was imaged every minute during the dose-response and stretch, except the first minute of stretch where the airways were imaged every second (Fig B in S1 File). From the acquired images, we quantified airway luminal area (Image J, NIH), and normalized its magnitude to the pre-treatment baseline value.

### Zyxin expression in human bronchi

Protein expression of Zyxin (ZYX) was measured by immunohistochemistry on formalin fixed paraffin embedded (FFPE) sections from two groups of subjects; the first group was used for discovery and validation of the gene expression results in ASM. Tissues for the first group were obtained through the International Institute for the Advancement of Medcine (IIAM, Edison, NJ, USA) and informed consent was obtained from next of kin for use of the tissue for research on molecular determinants of asthma. The study is approved by the Providence Health Research Ethics Board (H13-02173) (University of British Columbia, Vancouver, British Columbia, Canada). [53].

Tissues from the second group were obtained at post-mortem from a prospective study of airway structure in fatal and nonfatal asthma from Perth, Western Australia and from the Prairie Provinces Asthma Study, a multicenter study of asthma fatalities occurring in Alberta, Saskatchewan, and Manitoba Canada [54]. Informed consent was obtained from the next-of-kin for pathological studies of lung tissues. Their use for the study of the pathology of asthma was approved by the Sir Charles Gairdner Hospital (Western Australia) and the University of Calgary Ethics Committees. Subject characteristics can be found in Table A in S1 File.

### Subject selection and RNA isolation

Lungs from 12 asthmatics and 12 control subjects were used for RNA isolation. Non-asthmatic donor deaths were primarily due to head trauma while 8 of the 12 asthmatic subjects died during exacerbations of their asthma. Mechanical properties of the tracheal muscle from the donor lungs were measured as previously described [40]. Twenty frozen sections (10uM) from two samples from each lung were used for isolation of mRNA and morphometric assessment of airway remodeling.

### Nanostring gene expression

mRNA for zyxin and housekeeping genes was measured using a custom codeset panel and the Nanostring nCounter system. Data were normalized in accordance with Nanostring’s guidelines.

### Immunohistochemsitry

Protein expression of Zyxin (ZYX) was validated by immunohistochemistry in formalin fixed paraffin embedded (FFPE) sections from each of the 24 subjects used for RNA analyses. Antibodies for ZYX (ab110202) were ordered from Abcam. Sections of 4μm thickness were cut from the lung cores. FFPE sections were deparaffinized in CitriSolv (Fisher Scientific) and brought to water using graded series of alcohol. Sections were then autoclaved (Bench Top Tuttnauer 2340M autoclave) on a 22-minute cycle in Citra buffer, pH6, and were then left to cool for 20 minutes. All the IHC staining was carried out on a Dako Autostainer Universal Staining System and all incubations throughout the staining run were carried out at room temperature. After cooling, the slides were loaded onto the Autostainer and incubated in Background Sniper (BS966: Biocare Medical; Concord, CA, USA) for 15 minutes to help reduce non-specific binding. The sections were incubated with the primary antibody at 1/200 dilution for 45 minutes. Detection was performed using the MACH3 Universal AP staining kit (Biocare Medical). Slides were covered with MACH3 Universal AP Polymer (M4U536) for 10 minutes each. Finally, the sections were incubated with Vulcan Fast Red as chromagen (Biocare, FR805) for 12 minutes and then counterstained with diluted Gill’s III Hematoxylin (SurgiPath) for 2 minutes. As negative control, normal mouse serum was used at the same concentration as the primary antibody. Degree of staining was quantified for the whole airway, ASM bundles and epithelial layer. Quantification of the staining was done using the positive pixel count algorithm that is part of the Aperio Image Scope software. The area of ASM was traced to confine the algorithm to the correct tissue compartment. The algorithm variables, which were set to minimize non-specific identification of staining, were: Hue– 0.98, Hue width– 0.35, Saturation– 0.045. For each tissue compartment analyzed, the percent positivity and intensity were recorded. The percent positivity is the percentage of the traced area that is positive for the color of interest (in this case pink). A minimum of one slide per subject was stained for Zyxin. Subjects with more than one tissue section stained had their values averaged.

### Statistics

Unless otherwise noted, standard two-sided t-tests were used. Differences with p<0.05 were considered significant. For the studies of zyxin expression in human airway tissue, the normalized gene expression values for zyxin mRNA were compared between asthmatic and non-asthmatic subjects and between non-asthmatic, non-fatal asthmatic (n = 4) and fatal asthmatic subjects (n = 8) using a generalized linear model with a gamma distribution.

Since multiple batches of staining were necessary for completion of the study, percent positive data (percentage of area stained pink) was normalized to the positive control (Breast ductal tissuse). Data was not normally distributed so statistical analyses were performed on log-transformed values. Cohort 1 consisted of tissue collected in Vancouver from 12 asthmatics (4 non-fatal and 8 fatal) and 12 non-asthmatics. An ANOVA was performed to determine statistically significant differences between the groups. The larger cohort 2 consisted of tissue collected in Perth and Calgary from 43 asthmatics (21 non-fatal and 22 fatal) and 27 non-asthmatics. Preliminary data analysis identified a small but significant difference in staining between the two collection sites (Australia vs. Canada) and so an ANCOVA (analysis of co-variance) was performed with collection site acting as a co-variate in cohort 2. A p-value less than 0.05 was considered statistically significant.

## Results

**Fig 1:**
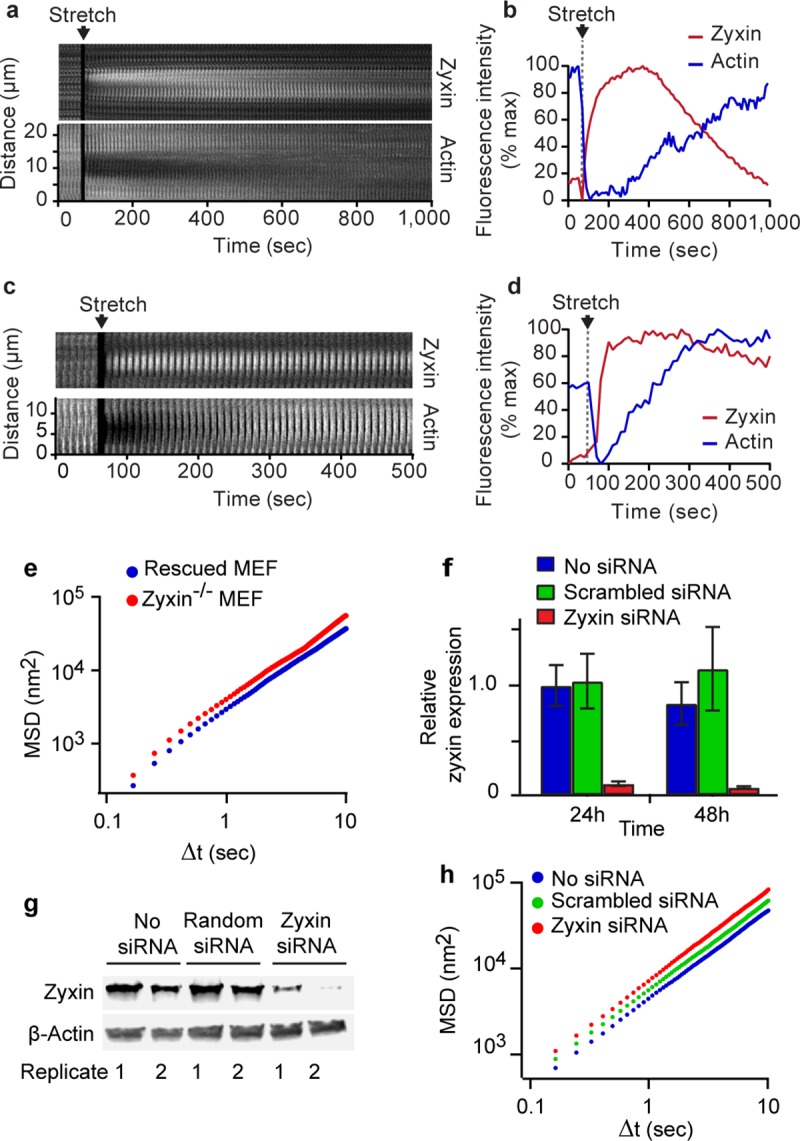
Zyxin is recruited to sites of actin stress fiber strain following single isotropic stretch, and is responsible for decreasing the cytoskeleton remodeling rate. (A) Kymographic analysis of zyxin and actin on a representative stress fiber following a single isotropic stretch. The kymograph is a time lapse montage of images of an actin SF captured in a zyxin^-/-^ MEF rescued with zyxin-GFP and also expressing actin-mApple Zyxin localizes to sites of stress fiber fragmentation and facilitates stress fiber repair. (B) Quantification of actin and zyxin intensity on a representative stress fiber following a single isotropic stretch showing zyxin’s localization to a site of stress fiber fragmentation and the subsequent recovery of the stress fiber. Kymograph (C) and intensity analysis (D) of zyxin recruitment to an actin strain site in a HASM cells. (E) Mean square displacements (MSD) of microbeads adherent to the cytoskeleton in zyxin^-/-^ and GFP-zyxin-rescued MEFs. Zyxin^-/-^ cells have an increased rate of remodeling, as evidenced by the upward shift of the MSD curve. (F) qPCR shows successful knockdown of zyxin in HASM cells of approximately 90% by 48 hours. (G) Western blot shows large reduction in zyxin in HASM cells by 72 hours post-transfection. Two replicates of this knockdown are shown as the two lanes for each condition in the western blot. (H) Cytoskeletal remodeling rate in HASM cells is increased after zyxin knockdown as indicated by the upward shift of the MSD curve.

Zyxin^-/-^ mouse embryo fibroblasts (MEFs) were rescued with exogenously expressed zyxin fused with green florescence protein (zyxin-GFP), and transfected with Lifeact-mApple to track actin dynamics[43]. To simulate a DI, we subjected cells co-expressing both fluorescent proteins to a stretch and release of 10% strain amplitude and 4s duration[16, 22]. At baseline, zyxin was predominantly localized at focal adhesions (S1 Movie). Within 30 seconds of the stretch and release, zyxin began to localize to sites of actin fragmentation, as identified by local decreases in Lifeact intensity along stress fibers, and persisted on stress fibers for at least five minutes (Fig 1A and 1B, S1 Movie and S2 Movie). These data are consistent with the timescale of zyxin-dependent repair of actin strain sites reported previously, and also with the timescale of airway smooth muscle (ASM) resolidification after stretch-induced fluidization[21, 22]. To test whether the phenomenon of stretch-induced recruitment of zyxin to sites of acute actin strain was generalizable to other cell types, we performed similar experiments in primary human ASM (HASM) cells. Zyxin was recruited with similar dynamics in these cells (Fig 1C and 1D). Together, these data indicate that the rapid zyxin response to physiologically relevant stretch occurs not only in MEFs but also in primary HASM cells.

Previous work has shown that zyxin^-/-^ cells fail to reinforce actin stress fibers when subjected to uniaxial cyclic stretch [45], thus supporting the hypothesis that one critical role for zyxin is to stabilize the cell mechanically. To examine remodeling dynamics in cells with and without zyxin, we tracked the movement of microbeads coupled to focal adhesions through a coating of the integrin-binding peptide sequence RGD [48]. These beads bind through focal adhesions to major stress fibers [55]. Because an attached microbead cannot move spontaneously unless the associated stress fiber remodels, spontaneous molecular-scale motions of an attached bead have been taken as a reporter of the rate of internal cytoskeletal reorganization [21, 48]. In MEFs, these micro-scale bead motions were 45% larger in amplitude in zyxin^-/-^ cells compared with beads in zyxin-GFP rescued cells (Fig 1E). A similar result was obtained in primary HASM cells treated with zyxin siRNA. Transfection of zyxin-specific siRNAs in the HASM cells led to 91± 2% reduction in zyxin mRNA at 48 hours (Fig 1F), and 88 ± 10% reduction in the zyxin protein levels at 72 hours (Fig 1F and 1G). Zyxin knockdown in HASM cells increased the rate of spontaneous microbead movement by 33% compared with HASM cells treated with non-targeting siRNA (Fig 1H). These results indicate that the rate of cytoskeletal remodeling is zyxin-dependent, consistent with the hypothesis that zyxin alters actin dynamics and actively stabilizes cell cytoarchitecture.

**Fig 2:**
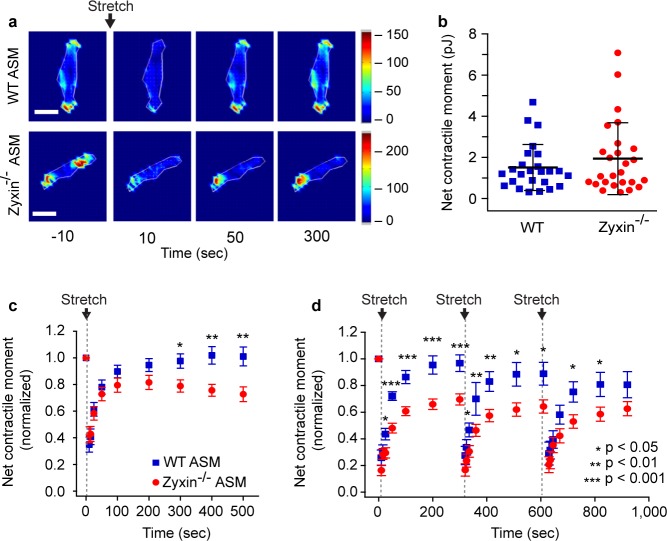
Zyxin mediates post-fluidization resolidification in MASM. (A) Representative traction maps of wild type and zyxin^-/-^ primary ASM cells at baseline and 10, 50 and 300 seconds after a single transient isotropic stretch. Maps show colorized distribution of forces. The legend shows the color values in KPa. (B) Net contractile moment is similar in both zyxin^-/-^ and wild type primary MASM cells in isometric conditions. (C) Normalized changes in net contractile moment in wild type (n = 25) and zyxin^-/-^ (n = 26) primary MASM cells following a transient 5–10% stretch at time = 0 seconds. (D) Normalized changes in net contractile moment in wild type (n = 22) and zyxin^-/-^ (n = 23) primary ASM cells following a series of three transient 5–10% stretches at 0, 310 and 620 seconds.

The rapid localization of zyxin to sites of stress fiber strain and its role in actin remodeling [41], repair [43] and stabilization raises the possibility that zyxin might be important in stretch-induced cytoskeletal recontraction in ASM cells [22]. In the lung during normal physiological conditions, ASM cells ordinarily strain by about 4% of their length with each tidal breath and by as much as 25% of their length with each spontaneous DI, which occur in human adults at a frequency of approximately once every 6 minutes [56] [5, 16]. Experimental maneuvers in culture can recapitulate stretch dynamics that occur during breathing and can be monitored simultaneously using traction force microscopy[49] to measure contractile forces before and after a single isotropic stretch and release (Fig 2A). To examine the role of zyxin in this process, we isolated primary ASM cells from zyxin^-/-^ and wild type (WT) mice. Western blot analysis confirmed the absence of zyxin protein in the zyxin^-/-^ cells, as well as abundant zyxin protein expression in the WT MASM cells (Fig A in S1 File). Additionally, western blotting was used to check the levels of other proteins involved in cell contractility and stress fiber organization, including α-actinin, β-actin, CRP1, myosin, and VASP with no significant changes observed (Fig A in S1 File).

In zyxin^-/-^ versus WT primary MASM cells, we observed no difference in the isometric contractile force prior to stretch (Fig 2B). Immediately following a single transient stretch (5–10% strain), both cell types relaxed rapidly and the net contractile force measured by traction microscopy [49] decreased by 65 ± 6% for WT and 58 ± 6% for zyxin^-/-^, which was not statistically significant (Fig 2B and 2C). However, whereas in WT cells the contractile force recovered over the next 300 seconds to 101 ± 7% of baseline, zyxin^-/-^ cells failed to recontract at the same rate and to the same extent so that by 500 seconds post-stretch, recontraction of zyxin^-/-^ cells had plateaued and attained only 73 ± 6% of baseline (p<0.01)(Fig 2B and 2C). To simulate physiologic conditions even more closely, we repeated stretch and release approximating the frequency of DIs observed during spontaneous breathing [5]. Within the 300 seconds following each stretch, wild type MASM cells recovered to 81–97% of their baseline initial contractile force whereas zyxin^-/-^ MASM cells recovered to only 63–70% of pre-stretch contraction (p<0.05)(Fig 2D). These results show that in primary MASM cells following transient stretch, the rapid relaxation is unaffected in the absence of zyxin, but both the rate and the extent of recontraction are zyxin-dependent.

**Fig 3:**
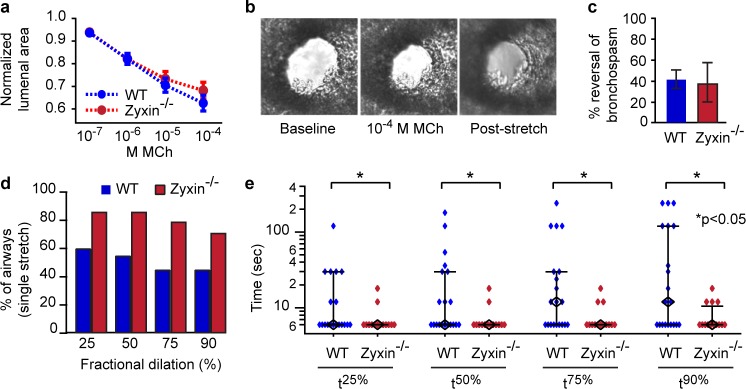
Airways in zyxin^-/-^ PCLS show similar agonist response but exhibit more rapid dilation following stretch. (A) Normalized changes in lumenal area of airways from wild type (n = 114 airways) and zyxin^-/-^ (n = 75 airways) animals in response to increasing doses of methacholine. B) Representative images of an airway initially, after methacholine treatment and following 3 minutes of stretch. (C) No significant differences in the magnitude of reversal of bronchospasm were observed between airways from wild type (n = 20 airways) and zyxin^-/-^ (n = 14 airways) animals. (D) Percentage of airways reaching a given fraction of their ultimate steady state dilation with a single stretch. (E) Times to achieve 25%, 50%, 75%, and 90% of final steady state dilation (black diamonds are median values, whiskers are IQR). (*p<0.05).

To look more closely at the cellular response in intact murine airways, we assayed precision cut lung slices (PCLS) [15, 50] obtained from WT and zyxin^-/-^ mice. We contracted airways embedded within lung parenchyma by incubating the PCLS in methacholine (MCh) in the absence of stretch, and then stretching the PCLS, along with its embedded airways, using a punch-indenter system [15] (Fig B in S1 File). As the concentration of MCh was increased, airway lumenal area decreased in both WT and zyxin^-/-^ airways (Fig 3A). When challenged with 10^−4^ M MCh, the WT airways showed a mean decrease in airway lumenal area of 37 ± 3%, which is consistent with previous studies in C57BL/6 animals [57] and was not significantly different from the decrease in lumenal area of 32 ± 4% observed in zyxin^-/-^ airways (n = 114 WT, 75 zyxin^-/-^ airways, p = 0.12)(Fig 3A).

Consistent with previous studies [15, 52, 57], responses to airway stretches within the PCLS varied from airway to airway in a manner that was highly variable, quite skewed, and did not follow a normal distribution. As such, to compare normalized changes of airway lumenal area between wild type PCLS versus zyxin^-/-^ PCLS, we used the Mann-Whitney-Wilcoxon rank-sum (MWW) test. In spite of the large variability noted above, statistically significant differences were noted. Although the average reversal of lumenal area by DIs was the same in the two strains, 40 ± 11% in WT airways (Fig 3B and 3C) and 37 ± 14% in zyxin^-/-^ airways (p = 0.3)(Fig 3C), zyxin^-/-^ airways achieved this dilation much more rapidly. For example, 79% of the zyxin^-/-^ airways dilated with the very first stretch to 75% of the final steady state area, whereas only 45% of WT airways did so (p = 0.05)(Fig 3D). The median time required for each airway to dilate to 90% of its final steady state area (t^90%^) was 50% less in zyxin^-/-^ versus WT airways (p<0.05)(Fig 3E), and times to reach t^25%^, t^50%^, and t^75%^ were also significantly lower in zyxin^-/-^ airways (p<0.05)(Fig 3E). Thus, in intact airways, the absence of zyxin did not change the extent of airway lumen recovery following a sequence of DIs, but substantially accelerated the time to dilation, suggesting a role for zyxin in stabilizing the contracted state of the airway.

**Fig 4:**
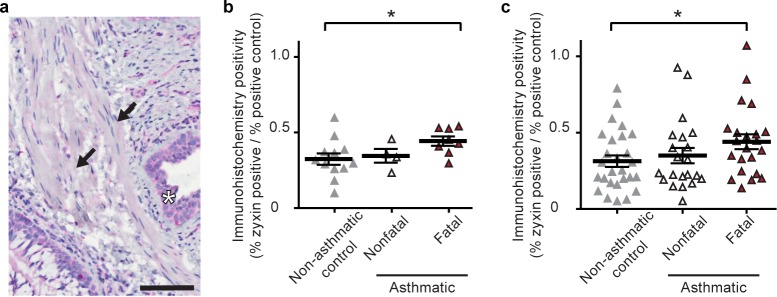
Quantification of zyxin protein expression in the airways of asthmatic and non-asthmatic bronchi. A) Representative image of zyxin IHC staining. Black arrow indicates ASM bands and black star indicates epithelial layer. Scale bar is 100um. B) Comparison of the percent positivity for zyxin in the ASM layer of asthmatics and non-asthmatics expressed as a fraction of the positive control. C) Comparison of percent positivity for zyxin in the ASM of non-asthmatic controls (n = 27), non-fatal asthmatics (n = 21) and fatal asthmatics (n = 22) in the replication cohort expressed as a fraction of the positive control. p<0.05 denotes significance.

These findings suggest that zyxin plays an important role in regulating recontraction in ASM cells after a DI and in stabilizing the contracted state prior to a DI. However, it remains unclear if zyxin actually plays a role in human asthma. To determine the localization of zyxin protein and its relative abundance in distinct airway wall compartments, we performed immunohistochemical staining in human tissues (Table 1). Diffuse staining for zyxin was evident in ASM, epithelial cells and inflammatory cells within the airway wall (Fig 4A). There was significantly greater zyxin staining in the ASM of people who died from asthma than in non-asthmatics and in asthmatics who died from other causes (p< 0.05)(Fig 4B). By contrast, we found no significant difference in zyxin staining in the airway epithelium or other non-ASM compartments in non-asthmatics, fatal asthmatics or non-fatal asthmatics. In addition, there were no significant differences between these groups in the amount of zyxin transcript as measured by qRT-PCR Nanostring assay (Fig C in S1 File). To confirm that zyxin expression is upregulated in the ASM of fatal-asthmatics compared with non-asthmatics, tissue from a second, larger replication cohort (Table A in S1 File) was stained with the same antibody and quantified in the same manner. These results confirmed that zyxin protein expression was significantly greater in the ASM of fatal-asthmatic subjects (n = 22) compared with non-asthmatic subjects (n = 27 p<0.05)(Fig 4C). In both cohorts, ASM zyxin staining in asthmatics who died of other causes (n = 4 in cohort 1 and 25 in cohort 2) was comparable with that of the non-asthmatics (p> 0.05)(Fig 4C). There was no relationship between zyxin staining and subjects’ age, smoking history, duration of asthma or use of inhaled corticosteroids, or as a function of airway size, measured by basement membrane perimeter.

**Table 1:** Subject characteristics for both initial discovery cohort and replication cohort. Age is expressed in years ± SD.

	Cohort 1			Cohort 2		
	Non-Asthmatic	Non-Fatal Asthmatic	Fatal-Asthmatic	Non-Asthmatic	Non-Fatal Asthmatic	Fatal-Asthmatic
Mean Age	27.8 ± 17.7	23.3 ± 10.3	16.0 ± 6.5	42.0 ± 19.7	33.3 ± 17.0	39.4 ± 20.2
Median Age	21	23	15	40	29	35
% Male	50	75	50	67	52	59
N	12	4	8	27	21	22

## Discussion

ASM possesses force-generating capacity that is sufficient to close virtually every airway in the intact lung [58] and therefore exhibits substantial capacity to impair physiological function in asthma [1–3, 9]. To prevent excessive airway narrowing when inflammatory, irritant, or neural events activate ASM, some mechanism must act to attenuate these smooth muscle forces. In the healthy lung, the dynamic loading induced by DIs and the resulting stretch of ASM fulfill this role and lead to dynamic equilibration of airway caliber [2–4, 14] [14] [15, 23].

The response of ASM to stretch is marked by disruption of actin-myosin interactions[16] and other weak bonds together with disruption of actin stress fibers (SFs), with resulting reduction of cellular contractile force and stiffness [21, 22]. This prompt relaxation response is followed by a slow and ATP-dependent recontraction wherein cellular contractile force, stiffness and structure return slowly to pre-stretch levels [21, 22, 59]. Slow and ATP-dependent recontraction necessarily encompasses reorganization, repolymerization and adaptation of the acto-myosin contractile apparatus and its actin cytoskeletal scaffolding and focal adhesions [21, 36, 59, 60] and thus implies active reassembly. Clearly, molecular regulators of this reassembly and its rate of progression could be therapeutic targets because, were recontraction to be blocked, cytoskeletal components of ASM would be unable to reassemble and contract, and bronchospasm would therefore be attenuated. However, specific regulators of reassembly had remained to be identified. Here we demonstrate an essential role for zyxin, which is seen to stabilize ASM cyto-architecture and contractile function at levels of cytoskeleton, cell, and tissue. Importantly, we have also found increased zyxin protein expression in the airways of patients who died of asthma.

In response to a single isotropic stretch simulating a DI, stress fiber fragmentation results in a profound drop in contractile force whereupon the zyxin mediated repair complex is rapidly recruited to sites of acute stress fiber fragmentation (Fig 1A and 1B). Wild type ASM cells are able to quickly repair the fragmented stress fibers, and, as a result, wild type cells are able to return to baseline levels of contractile force. On the other hand, zyxin null ASM cells, being unable to rapidly repair sites of stress fiber fragmentation, are unable to restore baseline levels of contractile force (Fig 3C and 3D and Fig D in S1 File). At the level of intact murine airways embedded within PCLS we observe this same phenomenon in a different context; we find a substantial increase in the rate of airway dilation following a simulated DI in an agonist contracted zyxin^-/-^ airway (Fig 4D and 4E). This finding is consistent with the observation that the stress fibers in chronically stretched zyxin null cells are significantly less robust that those in wild type cells. In summary, the loss of zyxin function in MASM cells and in isolated mouse airways results in decreased contractility following a DI. When we quantified zyxin levels in the ASM of asthmatics, we found the flip side of this loss of function phenotype; in fatal asthmatics, but not in non-fatal asthmatics, zyxin levels were significantly increased (Fig 4). Such an increased abundance of zyxin in fatal asthmatics who have refractory airway narrowing is consistent with the dynamic action of zyxin in promoting the reinforcement and resulting resolidification of ASM.

Based upon our structural and biophysical findings *in vitro* at the levels of cytoskeleton, cell and tissue, we expected that that increased accumulation of zyxin in fatal asthma might result in an overly reinforced cytoskeleton, excessive cytoskeletal resolidification and contraction, and, as a result, compromised ability to relax ASM in response to a DI. Consistent with that expectation, Chin et *al*. [40] showed that ASM from 6 asthmatics (4 fatal) versus 6 non-asthmatics has less relaxant response to a simulated DI and recovered faster following stretch. Ijpma et al.[61], by contrast, used a protocol similar to that of Chin et al. [40] but did not find a difference in the ASM response to stretch in asthmatics versus controls. However, 7 of 8 asthma cases in the study Ijpma et al were non-fatal [61] compared with 4 of 6 in the study of Chin et *al*. [40]. In the limited context of responses to stretch in ASM from non-fatal asthmatics, the observations reported here, by Chin et *al*. [40], and by Ijpma *et al*.[61] are mutually consistent.

The changes in zyxin localization at the subcellular level are rapid and transient. Chronically or acutely stretched cells reinforce their cyto-architecture through zyxin recruitment, but this accumulation of zyxin dissipates rapidly once the actin structures had been reinforced. Biophysical observations at the levels of cytoskeletal structure, single cell mechanics, and intact airways embedded within lung slices clearly establish that zyxin stabilizes the ASM contractile apparatus, its cytoskeletal scaffolding, and thus sustains the effect of ASM contraction on airway narrowing (Fig D in S1 File).

Observations in human tissue samples establish a clear increase in zyxin protein in the ASM of people who died as the result of an asthma attack (Fig 4). As such, zyxin is seen not only to reinforce airway smooth muscle but also to accumulate in airways of fatal asthmatics. Although these data implicate zyxin in the impaired ability of a DI to relax ASM in life threatening asthma [40], they do not determine whether high zyxin levels are a contributing cause or an effect of fatal asthma.

## Supporting information

S1 File **Fig A in S1 File. Primary ASM from zyxin**^**-/-**^ **mice express no zyxin.** (A) Western blot of wild type and zyxin^-/-^ primary ASM cells showing the absence of zyxin expression in zyxin^-/-^ cells. (B) Candidate regulators of contractility and stress fiber function do not show altered levels of expression in zyxin^-/-^ cells. **Fig B in S1 File. Applying stretch to the murine PCLS.** (A) Schematic of the annular indenter used to stretch PCLS (not to scale). (B) Cut-away view of the indenter showing the isotropic stretch of the gel and PCLS. (C) Experimental timeline of MCh dose-response and stretch, red bars indicate image acquisition. **Fig C in S1 File. Comparison of normalized transcript counts of zyxin measured by Nanostring nCounter system**. Comparisons were made between non-asthmatic controls (NAC,n = 12), non-fatal asthmatics (NFA, n = 4), fatal asthmatic (FA, n = 8) and all asthmatics (A, n = 12). **Fig D in S1 File. In response to a transient stretch mimicking the effect of a DI, contracted ASM fluidizes rapidly and then resolidifies slowly.** Zyxin acts dynamically during the resolidification response to stabilize the contractile apparatus and its actin scaffolding at the levels of the SF, the isolated cell, and the integrated airway. (A) At the molecular level (top panel), zyxin acts to stabilize actin SFs, thereby inhibiting CSK remodeling. At the cellular level (middle panel), net contractile force in isometric conditions and the rapid fluidization in response to a DI are independent of the cytoskeletal protein zyxin, whereas the slow resolidification response is dependent upon zyxin. At the integrated tissue level (bottom panel), zyxin similarly stabilizes the contractile apparatus in ASM, slowing airway dilation from DI. Across these multiple scales of length, zyxin acts dynamically to promote cytoskeletal stabilization and resolidification. (B) At the cellular level (top panel), under isometric conditions zyxin is largely localized to cell adhesions. In response to an external mechanical stress, the cell fluidizes and zyxin localizes to sites of SF strain to facilitate their repair, stabilizing the contractile apparatus to again support high traction force. At the integrated tissue level (bottom panel), in response to mechanical stress, zyxin similarly stabilizes the contractile apparatus in ASM, slowing airway dilation from DI. **Table A. Subject characteristics for second cohort.**
(DOCX)

S1 Movie Timelapse micrograph showing a zyxin-/- mouse embryonic fibroblast transfected with zyxin-GFP and Lifeact-mApple undergoing a transient stretch and release of 10%. (MOV)

S2 Movie Zoomed region of S1 Movie. (MOV)
