# Supplementary material for: The actin regulator zyxin reinforces airway smooth muscle and accumulates in airways of fatal asthmatics
Source: PLoS One. 2017 Mar 9;12(3):e0171728. doi: 10.1371/journal.pone.0171728 (PMC5344679; doi:10.1371/journal.pone.0171728)
Supplement: S1 File — Fig A in S1 File. Primary ASM from zyxin-/- mice express no zyxin. (A) Western blot of wild type and zyxin-/- primary ASM cells showing the absence of zyxin expression in zyxin-/- cells. (B) Candidate regulators of contractility and stress fiber function do not show altered levels of expression in zyxin-/- cells. Fig B in S1 File. Applying stretch to the murine PCLS. (A) Schematic of the annular indenter used to stretch PCLS (not to scale). (B) Cut-away view of the indenter showing the isotropic stretch of the gel and PCLS. (C) Experimental timeline of MCh dose-response and stretch, red bars indicate image acquisition. Fig C in S1 File. Comparison of normalized transcript counts of zyxin measured by Nanostring nCounter system. Comparisons were made between non-asthmatic controls (NAC,n = 12), non-fatal asthmatics (NFA, n = 4), fatal asthmatic (FA, n = 8) and all asthmatics (A, n = 12). Fig D in S1 File. In response to a transient stretch mimicking the effect of a DI, contracted ASM fluidizes rapidly and then resolidifies slowly. Zyxin acts dynamically during the resolidification response to stabilize the contractile apparatus and its actin scaffolding at the levels of the SF, the isolated cell, and the integrated airway. (A) At the molecular level (top panel), zyxin acts to stabilize actin SFs, thereby inhibiting CSK remodeling. At the cellular level (middle panel), net contractile force in isometric conditions and the rapid fluidization in response to a DI are independent of the cytoskeletal protein zyxin, whereas the slow resolidification response is dependent upon zyxin. At the integrated tissue level (bottom panel), zyxin similarly stabilizes the contractile apparatus in ASM, slowing airway dilation from DI. Across these multiple scales of length, zyxin acts dynamically to promote cytoskeletal stabilization and resolidification. (B) At the cellular level (top panel), under isometric conditions zyxin is largely localized to cell adhesions. In response to an exter [file pone.0171728.s001.docx]

**Supporting information-S1 File**

**Fig A**

A
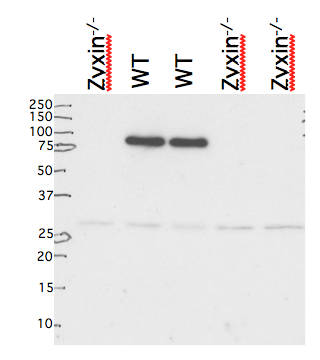


B
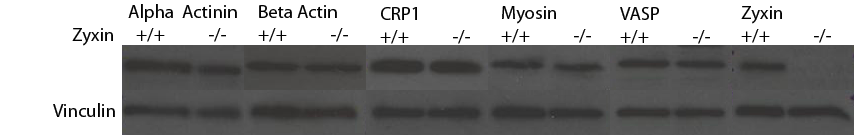


**Fig B**


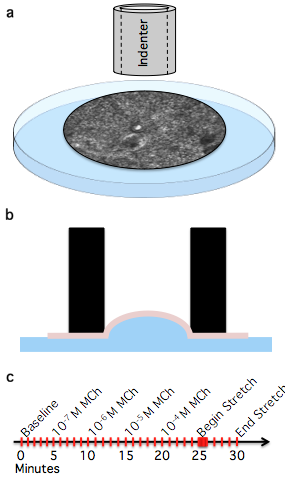


**Fig** C

**Fig D**


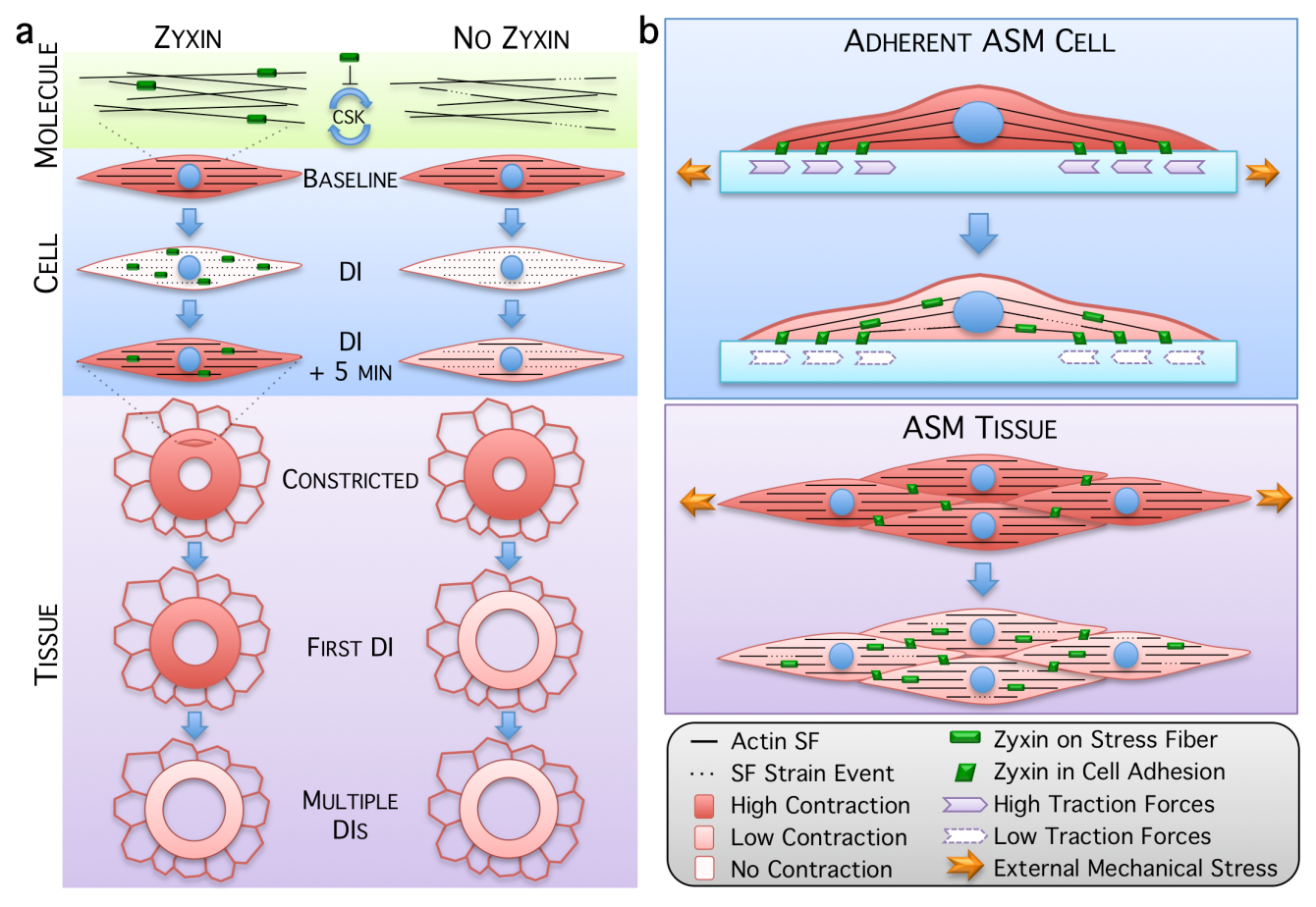


**Table A**

| **Gender** | **Age** | **Weight (kg)** | **Height (cm)** | **Ethnicity** | **Cause of Death** | **Medical History** | **Known Medication** | Terminal Medication |
| --- | --- | --- | --- | --- | --- | --- | --- | --- |
| *Discovery Cohort – Non-Asthmatics* | | | | | | | | |
| M | 20 | 86 | 185 | Caucasian | Head Trauma, MVA | Cigarettes 2/week; Vodka half bottle/week; Marijuana weekly | None | Vasopressors |
| F | 4 | 17.5 | 104 | Hispanic | Head Trauma | None | None | Steroids and vasopressors |
| M | 22 | 112 | 193 | Caucasian | Head Trauma | Occasional marijuana | None | Vasopressors |
| F | 63 | 77 | 159 | Caucasian | Gastrointestinal Bleed | Hypertension for 2 years | Pain medications, Antihypertensive | Antihypertensive |
| M | 14 | 50 | 165 | Caucasian | Head Trauma, MVA | None | None | Vasodilator |
| F | 19 | 87 | 165 | Caucasian | Head Trauma, MVA | Beer/Hard Liquor 1-2x/month for 2 years | None | Steroids and vasopressors |
| F | 20 | 72.2 | 165 | Caucasian | Head Trauma | Cigarettes <1PPD for 2 years, Marijuana | Inhalents, Pain medication | Vasopressors |
| M | 24 | 81.8 | 175 | Hispanic | Head Trauma | Alcohol on weekends | None | Vasopressors |
| M | 12 | 48.1 | 160 | Caucasian | Head Trauma, MVA | None | None | Vasopressors |
| F | 42 | 90 | 178 | Caucasian | Head Trauma, MVA | Hypertension for 1 year | Antihypertensive | Steroids and vasopressors |
| F | 47 | 82 | 167.5 | Caucasian | CVA | None | None | Amiodarone |
| M | 47 | 105 | 167.5 | Caucasian | CNS Tumor | Heart Murmur | Vasopressors, Solumedrol | Albuterol |

| **Gender** | **Age** | **Weight (kg)** | **Height (cm)** | **Ethnicity** | **Cause of Death** | **Medical History** | **Known Medication** | **Terminal Medication** |
| --- | --- | --- | --- | --- | --- | --- | --- | --- |
| *Discovery Cohort – Asthmatics* | | | | | | | | |
| M | 11 | 65.3 | 152.5 | Caucasian | Head Trauma | Asthma diagnosed age 6 | None | Steroids and vasopressors |
| F | 21 | 55 | 170 | Caucasian | Drug Overdose (Tylenol) | Asthma; Cervical Cancer free 1 year; Cigarettes 2PPD for 6 years | Aspirin, Albuterol, Advair | N/A |
| F | 15 | 56 | 162.5 | Caucasian | Anoxia, Probable Asthma Attack | Asthma; cigarettes <1PPD for 1 year; Marijuana 2x/week | Singular, Advair, Claritin, Flovent, Albuterol, Ritalin, Solumedrol, Albuterol | Steroids and vasopressors |
| M | 11 | 69 | 170 | Caucasian | Anoxia, Probable Asthma Attack | Asthma diagnosed age 2 | Albuterol | Vassopressors |
| F | 26 | 91.6 | 168 | Caucasian | Anoxia, Probable Asthma Attack | Asthma since childhood; cigarettes 1 pack/week since 16; heavy drinker | Albuterol, Advair, Lorazepam, Xanax | Vassopressors |
| M | 10 | 43 | 157.5 | Caucasian | Asthma Attack | Asthma diagnosed age 4 | Advair, Allegra, Albuterol | Steroids and vasopressors |
| M | 25 | 103 | 185 | Hispanic | Anoxia, Suicide | Asthma diagnosed age 11; cigarettes 2 PPD 12 years; Marijuana 6x/day for 12 years | Albuterol, Advair, Naproxen and Trazodone | Steroids and vasopressors |
| F | 8 | 30.5 | 117 | Hispanic | Asthma Attack | Asthma diagnosed age 3. RSV age 2 | Albuterol, Singular, Rantadine | Steroids and vasopressors |

Table A.
